#  

**DOI:** 10.1002/rmb2.12436

**Published:** 2021-12-21

The publication of invaluable papers in the Reproductive Medicine and Biology depends on the prompt, careful review of submitted manuscripts. We would like to thank the Editorial Board members, the Editorial Advisory Board members and the following experts for reviewing manuscripts from November 1, 2020, to October 31, 2021.

Hidenori Akutsu

Ahmed T Alahmar

Hisao Ando

Yoshimasa Asada

Yoshiko Asai

Yoshiyuki Asai

Atsushi Azumaguchi

Tsuyoshi Baba

Koji Chiba

Keisuke Edashige

Noritoshi Enatsu

Toshiaki Endo

Ricardo Felberbaum

Shunsaku Fujii

Masakatsu Fujinoki

Aisaku Fukuda

Atsushi Fukui

Jun Hagiuda

Kenshiro Hara

Miyuki Harada

Shu Hashimoto

Kenichiro Hiraoka

Tetsuya Hirata

Yasushi Hirota

Kentaro Ichioka

Masashi Iijima

Masahito Ikawa

Bunpei Ishizuka

Nobuhiko Itami

Masahiro Itoh

Naoki Itoh

Shoichiro Iwatsuki

Shikha Jain

Seung Chik Jwa

Daisuke Kadogami

Takeshi Kajihara

Haruhiko Kanasaki

Satoru Kaneko

Yukiko Katagiri

Satoshi Kawachiya

Kazuhiro Kawamura

Yasushi Kawano

Khaleque Khan

Kazuhiro Kikuchi

Fuminori Kimura

Koji Kimura

Masayuki Kinutani

Hiroshi Kishi

Satoshi Kishigami

Michio Kitajima

Jo Kitawaki

Ryota Kobayashi

Kaori Koga

Shouhei Komemoto

Mitsuru Komeya

Shinnosuke Komiya

Masataka Kudo

Hiroki Kurahashi

Keiji Kuroda

Shin‐no‐suke Kuroda

Satoshi Kurosaka

Yoshimitsu Kuwabara

Hirohisa Kyogoku

Koichi Kyono

Jung Ryeol Lee

Eri Maeda

Ryo Maekawa

Hiroshi Masuda

Hidehiko Matsubayashi

Yasuyuki Mio

Katsuyuki Miura

Yasushi Miyagawa

Toshinobu Miyamoto

Yuki Mizuguchi

Satoshi Mizuno

Debbie Montjean

Taisuke Mori

Tetsunori Mukaida

Srabani Mukherjee

Motoo Nabeta

Masashi Nagano

Tatsuya Nakano

Yoshiharu Nakaoka

Akitoshi Nakashima

Kaei Nasu

Takuji Nishihara

Taichi Noda

Masanori Ochi

Akihide Ohkuchi

Hidetaka Okada

Hiroaki Okae

Osamu Okitsu

Masanori Ono

Makoto Orisaka

Satoko Osuka

Kuniaki Ota

Nobuaki Ozawa,

Rangsun Parnpai

Hiroshi Sasaki

Takeshi Sato

Takuya Sato

Makoto Seiki

Akihiko Sekizawa

Surendra Sharma

Koichiro Shimoya

Masahide Shiotani

Hiromitsu Shirasawa

Tetsuji Soda

Ryota Suganuma

Yodo Sugishita

Seido Takae

Toshifumi Takahashi

Yasushi Takai

Satoru Takamizawa,

Seiji Takashima

Kumiko Takeda

Teppei Takeshima

Isao Tamura

Masaki Tanaka

Fuminori Taniguchi

Hidetaka Tasaki

Aytekin Tokmak

Isao Tsuji

Hiroshi Uchida

Satoshi Ueno

Takashi Umehara

Osamu Wada‐Hiraike

Sayaka Wakayama

Hideaki Watanabe

Budi Wiweko

Mitsutoshi Yamada

Takahiro Yamada

Yuri Yamamoto

Yasuhisa Yamashita

Atsumi Yoshida

Manabu Yoshida

Osamu Yoshino

Yasushi Yumura

